# Associations between bone material strength index and FRAX scores

**DOI:** 10.1007/s00774-024-01575-7

**Published:** 2025-01-18

**Authors:** Pamela Rufus-Membere, Kara B. Anderson, Kara L. Holloway-Kew, Mark A. Kotowicz, Adolfo Diez-Perez, Julie A. Pasco

**Affiliations:** 1 Deakin University, IMPACT- Institute for Mental and Physical Health and Clinical Translation, School of Medicine, Geelong, Australia; 2 Barwon Health, Geelong, Australia; 3 Department of Medicine-Western Health, The University of Melbourne, St. Albans, Australia; 4 Department of Internal Medicine, Hospital del Mar-IMIM, Pompeu Fabra University, Barcelona, Spain; 5 Department of Epidemiology and Preventive Medicine, Monash University, Melbourne, Australia

**Keywords:** Impact microindentation, Fracture, FRAX, Bone material strength index, Osteoporosis

## Abstract

**Introduction:**

Impact microindentation (IMI) measures bone material strength index (BMSi) in vivo. However, its ability to predict fractures is still uncertain. This study aimed to determine the association between BMSi and 10 year fracture probability, as calculated by the FRAX algorithm.

**Materials and methods:**

BMSi was measured using the OsteoProbe in 388 men (ages 40–90 yr) from the Geelong Osteoporosis Study. The probabilities for a major osteoporotic fracture (MOF) and hip fracture (HF) were calculated using the Australian FRAX tool. Hip (HF) and major osteoporotic (MOF) fracture probabilities were computed with and without the inclusion of femoral neck bone mineral density (BMD). For each participant, four 10 year probability scores were therefore generated: (i) HF-FRAXnoBMD; (ii) HF-FRAXBMD; (iii) MOF-FRAXnoBMD; (iv) MOF-FRAXBMD.

**Results:**

BMSi was negatively correlated with age (*r* = − 0.114, *p* = 0.025), no associations were detected between BMSi and femoral neck BMD (*r* = + 0.035, *p* = 0.507). BMSi was negatively correlated with HF-FRAXnoBMD (*r* = − 0.135, *p* = 0.008) and MOF-FRAXnoBMD (*r* = − 0.153, *p* = 0.003).

These trends held true for HF-FRAXBMD (*r* = − 0.087, *p* = 0.094) and MOF-FRAXBMD (*r* = − 0.111, *p* = 0.034), but only the latter reached significance.

**Conclusion:**

BMSi captures the cumulative effect of clinical risk factors in the FRAX algorithm, suggesting that it could provide additional information that may be useful in predicting risk of fractures. Further studies are warranted to establish its efficacy in predicting fracture risk.

**Supplementary Information:**

The online version contains supplementary material available at 10.1007/s00774-024-01575-7.

## Introduction

It is well known that fragility fractures constitute a major public health concern, due to the morbidity, mortality, decreased quality of life and cost associated with fracture-related injuries [1–3]. In Australia, every second woman and fourth man aged 50 years and above will experience such a fracture [4]. Hence, fracture prevention is a critical goal in older adults. Dual-energy X-ray absorptiometry (DXA)-derived bone mineral density (BMD), the current gold standard for assessment of fracture risk, is however limited with respect to fracture prediction since the highest burden of fractures originates from people with moderate deficits in bone mass and, conversely, not all individuals with large deficits in bone mass experience fracture [5]. Other clinical risk factors such as age, body mass index (BMI), parental hip fracture, prior fracture, use of medications including oral glucocorticoids, smoking and falls have been identified as independent predictors of fracture risk [6]. Consequently, the integration of multiple risk factors for fracture into assessment tools such as FRAX [7], the GARVAN nomogram [8] and our own FRISK score [9] have helped improved fracture risk estimates and inform management decisions.

The importance of age-related changes to compact bone fragility has been known for a long time, but its clinical importance has recently become emphasised in relation to osteoporotic fracture [10]. Damage caused by microcracks compromises bone strength [10, 11] but this is not fully captured by conventional BMD measurements [11]. A relatively novel device, OsteoProbe, assesses bone material strength index (BMSi), the resistance of cortical bone to microindentation [12, 13]. Some studies have indicated that patients with osteoporosis show significant changes in collagen cross-linking and matrix mineralisation that may contribute to skeletal fragility [14]. The cortical component is therefore an important determinant of bone strength, with increasing intra- cortical porosity and altered material properties of the matrix believed to contribute to bone fragility [15]. To date, studies have reported an association between low BMSi and cortical porosity [16], high prevalence of fracture [17], and its association [18, 19] or no association [20] with BMD. Rudang et al. further reported that BMSi did not improve fracture risk prediction in older women [20]. Hence, the ability of BMSi to discriminate fracture risk is still uncertain.

The aim of this study was to investigate associations between BMSi and risk of fracture, as evaluated using the 10 year fracture probability (FRAX), computed with and without the inclusion of femoral neck BMD. The FRAX tool computes fracture risk for people aged between 40 and 90 years based on models that incorporate the risks associated with clinical risk factors for fracture, and femoral neck BMD [7]. Given that BMSi is not or is weakly associated with age or BMD in most published reports, an association with FRAX would imply that BMSi may be capturing skeletal changes that relate to genetic factors, exposures and comorbidities included in FRAX, hence, may be clinically relevant in improving fracture risk predictions.

## Materials and methods

### Participants

Participants were from the Geelong Osteoporosis Study (GOS), a population-based cohort study situated in a geographically well-defined region in south-eastern Australia, known as the Barwon Statistical Division. Details of recruitment and retention have been described elsewhere [21]. The male arm of the GOS commenced in 2001 with recruitment of 1540 men aged 20–92 years. Participants are re-assessed every few years and data for this cross-sectional analysis were generated for 388 men assessed in 15 years follow-up phase (ages 40–90 years). The study was approved by the Human Research Ethics Committee at Barwon Health (00/56). All participants provided written informed consent.

### Impact microindentation (IMI)

Using the OsteoProbe RUO (Active Life Technologies, Santa Barbara, CA, USA), BMSi was measured on the anterior surface of the mid-tibia following standard guidelines [22]. The indentation site was determined by measuring the midpoint from the medial border of the tibial plateau to the distal edge of the medial malleolus. Following disinfection of the area and administration of local anesthetic, the OsteoProbe was inserted through the skin and periosteum until reaching the surface of the bone at the anterior face of the mid-tibia. At least 11 indentations were performed for each participant, of which the first measurement was systematically disregarded followed by 10 valid test indentations. The first measurement was disregarded to ensure sufficient penetration of the probe through the periosteum. Two trained operators conducted the IMI measurements (PR-M and KLH-K). The coefficient of variation (CV) for microindentation was 2% for repeated measures. Precision was calculated as the mean (expressed as %) of SD/mean for 2 sets of indentations for 10 participants in-vivo.

### Bone mineral density

Areal BMD (g/cm^2^) was measured at the femoral neck using DXA (Lunar, Prodigy, Madison, WI, USA). Quality control was maintained through weekly measurements of a Lunar DXA phantom. Height and weight were measured to the nearest 0.1 cm and 0.1 kg, respectively.

### Medical history, lifestyle factors and FRAX score

All participants completed comprehensive questionnaires detailing medical history, medication use and lifestyle behaviours. The FRAX probability for a major osteoporotic fracture (MOF) and for a hip fracture (HF) was calculated using the Australian FRAX tool [16]. For each participant, four 10 years probability scores were generated: (i) MOF-FRAXnoBMD; (ii) MOF- FRAXBMD; (iii) HF-FRAXnoBMD (iv) HF-FRAXBMD.

FRAX scores are based on risk factors for fracture including age, sex, weight, height, previous fracture, parental history of hip fracture, glucocorticoid use, rheumatoid arthritis, secondary osteoporosis, current smoking and alcohol use. Both MOF and hip fracture probabilities were computed with and without the inclusion of femoral neck BMD. A prior fracture was defined as any low trauma fracture equivalent to a fall from a standing height or less, excluding fractures of the toe, skull, finger and face, occurring during adulthood (age ≥ 20 years). Prior fractures were radiologically verified where possible; 70.8% of fractures were confirmed radiologically. A parental hip fracture referred to at least one maternal or paternal hip fracture. Current smoking referred to use of tobacco at least daily and excessive alcohol consumption as ≥ 3 units daily on average. Oral glucocorticoid use and rheumatoid arthritis were self-reported. Secondary osteoporosis including type 1 (insulin dependent) diabetes was determined, as previously described [23]. Osteogenesis imperfecta in adults, untreated long-standing hyperthyroidism and chronic liver disease were self- reported. A BMI < 18.5 kg/m^2^ (underweight) was used as a surrogate indicator of chronic malnutrition.

### Statistical Analysis

BMSi values were visually assessed for normality. Associations between BMSi values and FRAX scores as a continuous variables were identified using Pearson’s correlation. Secondary analyses investigated mean BMSi by FRAX cut-point. Based on the USA-adapted World Health Organization (WHO) treatment threshold (≥ 3% for HF and ≥ 20% for MOF) [24], FRAX scores were categorised into high and low. T-tests and box plots compare BMSi by FRAX category (both with and without BMD). Statistical analyses were performed using Minitab V.18 (State College, Pennsylvania, USA) and STATA Version 17.

## Results

### Characteristics

**Fig. 1:**
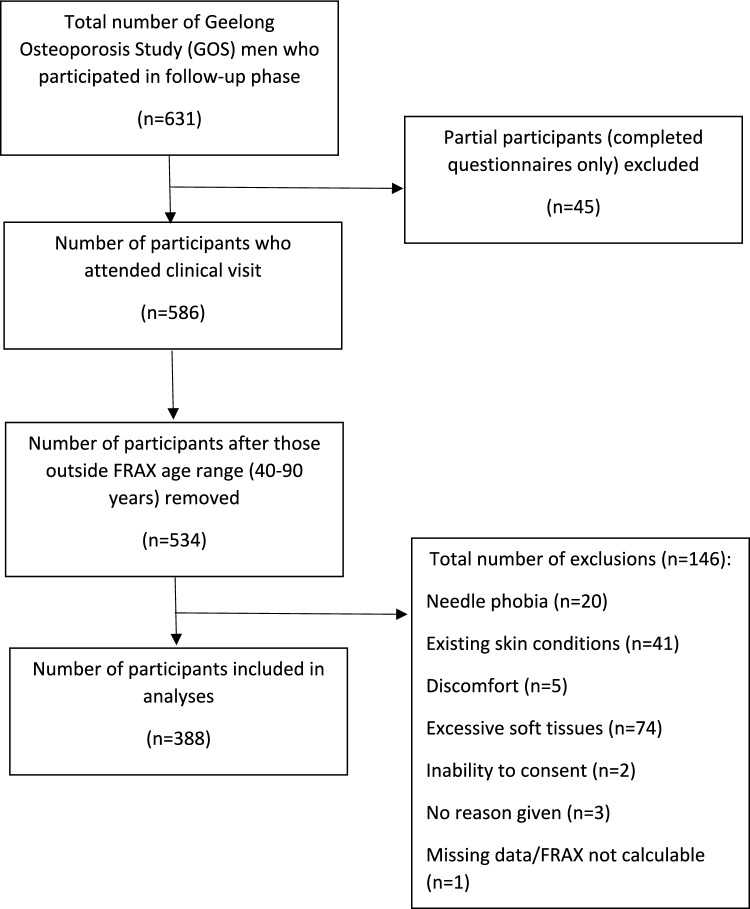
Flowchart showing the selection of participants included in the study

Of 535 potential participants in the current follow-up at time of writing, 389 underwent IMI testing (Fig. 1).

Reasons for non-measurement in 145 men were needle phobia (n = 20), existing skin conditions (n = 41), excessive soft tissues around mid-tibia region (n = 74), discomfort (pressure, not pain) after the first indentation (n = 5), inability to provide informed consent (n = 2) and three participants did not provide any reasons for declining. An additional participant was excluded from this participant due to insufficient data to calculate FRAX. Compared with participants, non-participants were older (70.3 ± 15.9 vs 64.2 ± 11.9 year, p < 0.001) and had greater mean BMI (30.2 ± 5.4 vs 27.0 ± 3.2, p < 0.001).

Participant characteristics and prevalence of risk factors are shown in Table 1. BMSi ranged from 49.0 to 99.4. Eighteen participants did not have femoral neck BMD measures, and thus only had FRAXnoBMD calculated. HF-FRAX and MOF-FRAX scores ranged from 0 to 17.0 and 0 to 24.0, respectively. BMSi was negatively correlated with age (r = − 0.114, p = 0.025). No associations were detected between BMSi and femoral neck BMD (r = + 0.035, p = 0.507). Additionally, participant characteristics by HF-FRAXBMD and HF-FRAXnoBMD categories are included in Supplementary Table 1.

**Table 1:** Participant characteristics (n = 388). Data are shown as mean ± SD, median (IQR) or n (%)

Age (yr)	63.0 (53.0–72.0)
Weight (kg)	82.2 ± 11.6
Height (cm)	174.4 ± 6.9
Body mass index (kg/m^2^)	27.0 ± 3.3
Femoral neck BMD (g/cm^2^)*	0.961 ± 0.13
Prior fracture^a^	75 (19.3)
Parental hip fracture	44 (11.3)
Smoking	26 (6.7)
Glucocorticoid use	7 (1.8)
Rheumatoid Arthritis	4 (1.0)
Secondary osteoporosis^b^	39 (10.1)
Alcohol consumption^c^	86 (22.2)

*missing data for eighteen men

^a^Fractures were: 7 vertebra, 6 hip, 4 foot, 9 elbow, 11 ankle, 5 humerus, 15 tibia and 18 rib

^b^Includes type 1 (insulin dependent) diabetes, untreated long-standing hyperthyroidism, osteogenesis imperfecta, chronic malnutrition and chronic liver disease

^c^Consumes 3 or more units of alcohol daily

### BMSi and FRAX Scores

Tables 2 (a), and (b) show results for associations between BMSi and FRAXnoBMD, and FRAXBMD, respectively. BMSi was negatively correlated with all FRAX indices (HF-FRAXnoBMD, MOF-FRAXnoBMD, HF-FRAXBMD and MOF-FRAXBMD), however correlations were stronger and statistical significance was reached at the < 0.01 level for FRAXnoBMD indices, whereas the trend did not reach this level of significance for FRAXBMD indices.

**Table 2:** Association between BMSi and Fracture risk (FRAX)-derived hip fracture parameters computed (**a**) without BMD and (**b**) with BMD

	Correlation	
	*r* value	*p* value
(**a**)		
HF-FRAXnoBMD	− 0.135	0.008
MOF- FRAXnoBMD	− 0.153	0.003
(**b**)		
HF-FRAXBMD	− 0.087	0.094
MOF- FRAXBMD	− 0.111	0.034

### High and Low FRAX Scores

**Fig. 2:**
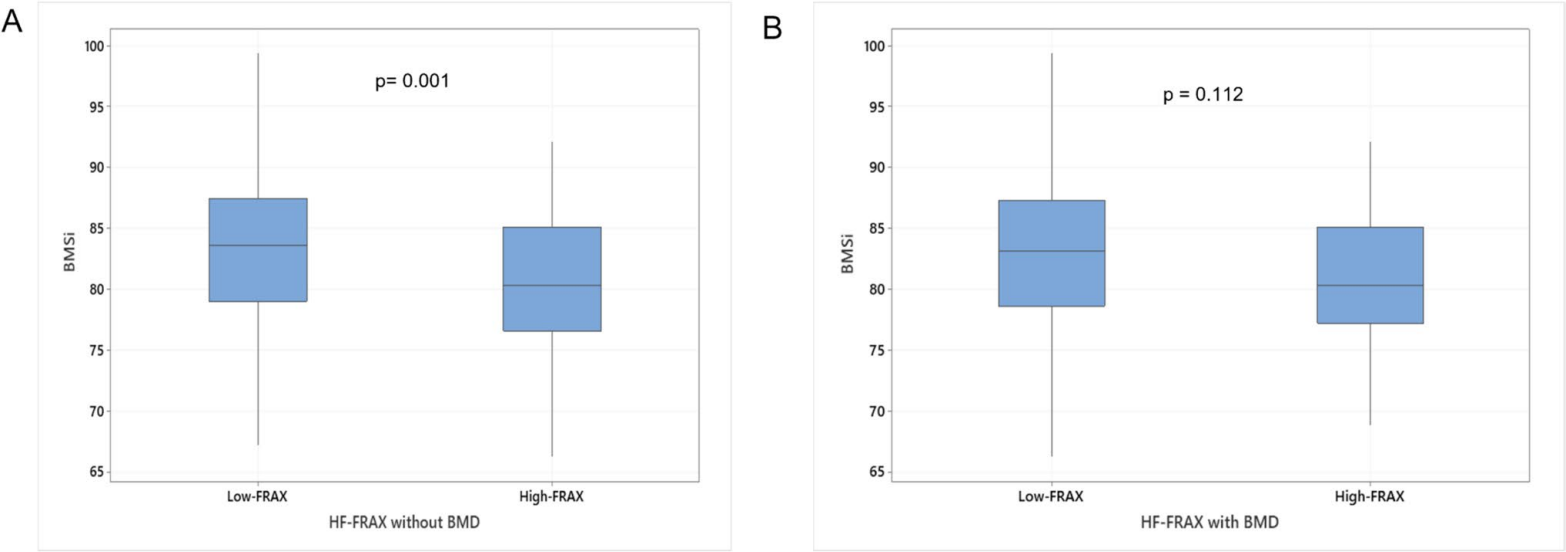
Boxplots showing bone material strength index (BMSi) and (**A**) HF-FRAX noBMD; **B** HF-FRAXBMD. Low FRAX is defined as a FRAX score < 3%, while High-FRAX is defined as a FRAX score ≥ 3

Based on the treatment threshold, 80/390 (20.5%) men had high HF-FRAXnoBMD, and 40/390 (10.3%) had high HF-FRAXBMD. There was a significant difference in mean BMSi between low and high HF-FRAX scores without BMD; 83.3 ± 7.5 vs 80.2 ± 7.5 (p = 0.001), but the difference between low and high HF-FRAX scores with BMD did not reach statistical significance; HF-FRAXBMD 83.0 ± 7.6 vs 81.0 ± 6.7 (p = 0.112) (Table 2, Fig. 2).

The numbers of men who exceeded treatment threshold for MOF- FRAXnoBMD (n = 1), and MOF-FRAXBMD (n = 0), were too small for subgroup analyses.

## Discussion

We report here that a higher BMSi was associated with a lower fracture risk, as calculated by the Australian FRAX algorithm. We also observed that the relationship between BMSi and calculated FRAX scores was weaker with the inclusion of BMD suggesting that BMSi was more strongly associated with FRAX scores when BMD was not considered. The inclusion of BMD dimishes the contribution of other clinical risk factors to the calculated FRAX scores, and BMSi may be more closely associated with these risk factors [25] or alternatively, when BMD is considered, the relationship is weaker because BMSi and BMD capture different elements of bone fragility. Our data complement and extend the only published study on the association between BMSi and FRAX scores by Malgo et al. in a study involving 90 male and female participants (ages 40.4–85.5 yr) with low bone mass with or without fragility fracture, in which they observed a negative correlation between BMSi and the 10-year fracture probability with and without the inclusion of femoral neck BMD [26]. Further, in this study, BMSi was negatively correlated with age, but no correlation was observed between BMSi and femoral neck BMD. To date, studies on the association between BMSi and age, and BMSi and BMD have shown conflicting results. For age, while Malgo et al. [27] reported BMSi was negatively correlated with age, Sosa et al. [19], and Popp et al. [28] found no association between BMSi and age. Additionally, we previously observed no association between age and BMSi in a subset of this cohort [29]. Notwithstanding, given that bone mass which is one of the major determinants of bone strength deteriorates with age [30], it seems reasonable that BMSi would decline with increasing age. The divergent results may be because of sample sizes, age ranges, and sampling frames of study participants. In relation to BMD, similar to our findings, Malgo et al.[18] and Sosa et al.[19] observed no association between BMSi and BMD, while Rudang et al. [20] observed a positive association between BMSi and BMD.

The results from this study suggest that BMSi might capture an element of the cumulative effect of risk factors in the FRAX algorithm to reduced material properties of bone, and consequently to increased fracture risk. This may partly be explained by the evidence that BMSi captures individual factors included in the FRAX algorithm. In an earlier study in this cohort, we reported that BMSi discriminates prior fracture and parental fracture [25]. Studies have also shown that mean BMSi is lower in groups with comorbidities known to affect fracture risk, such as, individuals with diabetes [31–37] or chronic kidney disease [38], and for those exposed to glucocorticoids [39].

A strength of this study is that we explored the relationship between BMSi and the risk of fracture, as calculated by the FRAX algorithm, in the largest sample covering the entire age range for FRAX (40–90 years). Unlike previous studies, this study is population-based and not selected based on disease status. Thus, the outcome will be applicable to the broader male population in this setting. Where possible, we have used objective measures for factors such as anthropometry and BMD; however, we also utilised some self-reported data, which may have been subject to recall bias. Moreover, these results are from largely Caucasian men and thus may not be relevant for other populations. In summary, our results indicate that increased risks of MOF and HF as estimated by FRAX are significantly associated with lower BMSi scores, independent of BMD. The information provided here is cross-sectional and lays the foundation for longitudinal studies that will improve the study of the value of BMSi in predicting fracture risk and ultimately, identify people who may benefit from early intervention.

## Supplementary Information

Below is the link to the electronic supplementary material.

Supplementary file1 (DOCX 108 KB)
