# Supplementary material for: Associations between bone material strength index and FRAX scores
Source: J Bone Miner Metab. 2025 Jan 18;43(3):230–6. doi: 10.1007/s00774-024-01575-7 (PMC12089204; doi:10.1007/s00774-024-01575-7)
Supplement: Supplementary file 1 — Supplementary file1 (DOCX 108 KB) [file 774_2024_1575_MOESM1_ESM.docx]

**Supplementary Table 1.** Participant characteristics (n=388) by HF-FRAXnoBMD and HF-FRAXBMD treatment threshold categories. Data are shown as mean ± SD, median (IQR) or n (%) as appropriate.


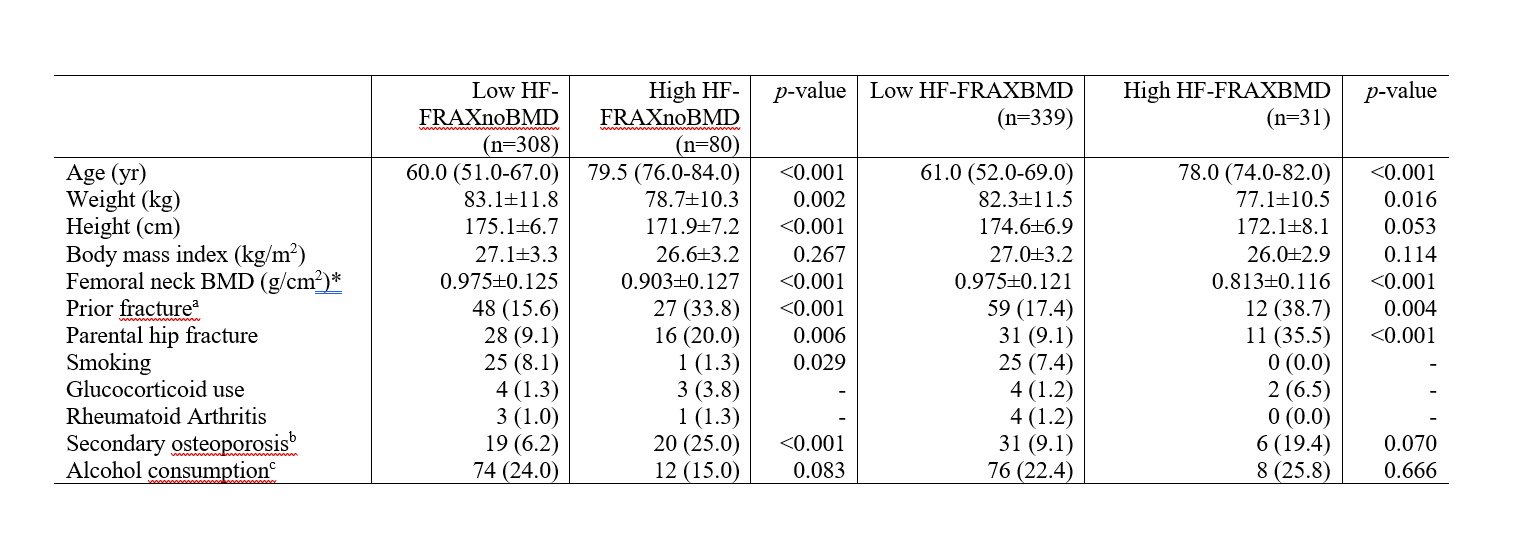


*- missing data for four men

1. fractures were: 7 vertebra, 6 hip, 4 foot, 9 elbow, 11 ankle, 5 humerus, 15 tibia and 18 rib.
2. includes type 1 (insulin dependent) diabetes, untreated long-standing hyperthyroidism, osteogenesis imperfecta, chronic malnutrition and chronic liver disease
3. consumes 3 or more units of alcohol daily
